# Integrated Transcriptomic Analysis Identifies Candidate Regulators Associated with Coconut Zygotic Embryo Development and Haustorium Maturation

**DOI:** 10.3390/plants15213461

**Published:** 2026-11-06

**Authors:** Zhiying Li, Yin Min Htwe, Dapeng Zhang, Peng Shi, Qun Yu, Xiangman He, Yong Wang

**Affiliations:** State Key Laboratory of Tropical Crop Breeding/Coconut Research Institute, Chinese Academy of Tropical Agricultural Sciences, Wenchang 571339, China; lizhiyingalien@gmail.com (Z.L.); yinminhtwemgk@gmail.com (Y.M.H.); zhangdp@catas.cn (D.Z.); ship@catas.cn (P.S.); yuqun1998@gmail.com (Q.Y.); 18389739413@163.com (X.H.)

**Keywords:** haustorium, phytohormones, transcriptome, transcription factors, zygotic embryo

## Abstract

Coconut zygotic embryo germination supports seedling establishment through coordinated embryo growth, tissue differentiation, and nutrient acquisition. A distinctive feature is the cotyledonary haustorium, which develops within the endosperm and transfers stored reserves to the growing seedling. However, transcriptional changes linking early germination to haustorium maturation and their relationships with phytohormone dynamics remain poorly understood. Here, we show that integrated time-resolved RNA sequencing, transcription factor (TF) and co-expression analyses, functional enrichment, and phytohormone profiling across 1–8 weeks after germination (WAG), including shoot/root (SR) and haustorium (Ha) tissues, reveal distinct developmental programs. We identified 792 core differentially expressed genes (DEGs), which formed four expression clusters. Cluster 4 was associated with early embryo development and included transcription factor (TF) modules involving *VP1*, *ABI4*, *ARF1*, *BBM2*, *TCP4*, *ERF003*, *GRF2*, and *GRF6*. In contrast, Cluster 1 increased during haustorium development and was enriched in hormone signaling, flavonoid biosynthesis, and cutin, suberine, and wax biosynthesis. *MIF1*, *HOX16*, and *CPC* showed high co-expression network connectivity. Hormone profiling showed relatively high auxin- and cytokinin-related metabolites early, progressive abscisic acid (ABA) increases, elevated ABA, indole-3-acetic acid (IAA), jasmonic acid (JA), jasmonoyl-isoleucine (JA-Ile), and salicylic acid (SA) in 6WAG-Ha, and increased methyl jasmonate (MeJA) and salicylic acid glucoside (SAG) in 8WAG-Ha. These findings reveal transcription factors and phytohormones during zygotic embryo germination, providing new understanding and reference to genetic transformation and somatic embryogenesis in coconut and other palm crops.

## 1. Introduction

Coconut (*Cocos nucifera* L.) is an economically important perennial palm whose seed contains a large endosperm reserve that supports embryo germination and subsequent seedling establishment. A distinctive feature of coconut germination is the development of a specialized cotyledonary haustorium, which expands within the endosperm cavity and facilitates the mobilization, absorption, and transfer of stored reserves to the growing seedling. This process involves coordinated transitions from early post-germination growth to shoot–root establishment and haustorium differentiation. Previous anatomical and biochemical studies have linked haustorium development to reserve utilization and lipid metabolism, highlighting its importance for successful seedling establishment [1,2]. At the molecular level, these developmental transitions are regulated by interconnected transcriptional and phytohormone networks. Auxin contributes to embryonic patterning, cell division, and tissue differentiation, whereas (ABA) regulates seed maturation, dormancy, and germination; GA, cytokinins, JA, and SA also contribute to developmental and stress-response processes [3,4,5,6,7]. Transcription factors (TFs), including BBM, GRF, and B3-domain family members, are important components of developmental regulatory programs that coordinate embryo growth and maturation [8,9,10].

Despite advances in understanding plant embryogenesis and hormone signaling, the molecular processes connecting early coconut zygotic embryo germination with subsequent tissue differentiation and haustorium maturation remain insufficiently characterized. Although transcriptomic and single-cell studies have begun to reveal developmental programs in coconut embryos and embryogenic tissues [8], the temporal changes in gene expression associated with haustorium development have not been comprehensively resolved across successive developmental stages. Furthermore, how candidate TF-associated expression programs relate to endogenous phytohormone dynamics during these transitions remains unclear. This knowledge gap limits the identification of candidate genes and regulatory modules associated with embryo establishment, haustorium differentiation, and reserve utilization.

We hypothesized that coconut zygotic embryo germination and haustorium maturation involve distinct, stage-specific transcriptional programs associated with dynamic changes in endogenous phytohormone profiles. To test this hypothesis, we integrated time-resolved RNA sequencing, DEGs analysis, TF identification, gene co-expression network analysis, functional enrichment, and phytohormone profiling across 1–4 WAG and in separate SR and Ha tissues at 6 and 8 WAG. Specifically, we aimed to identify core developmental DEGs, characterize their expression trajectories, identify candidate TF-associated modules linked to early development and haustorium maturation, and examine how phytohormone profiles change across developmental stages and tissues. By integrating transcriptional and hormonal evidence, this study provides a framework for understanding the molecular transitions accompanying coconut embryo germination and haustorium development and prioritizes candidate genes and pathways for future functional validation. These findings may also inform future efforts to improve coconut embryo culture and somatic embryogenesis, although the proposed regulatory relationships require experimental validation.

## 2. Results

### 2.1. Differentially Expressed Genes (DEGs) Analysis

**Figure 1:**
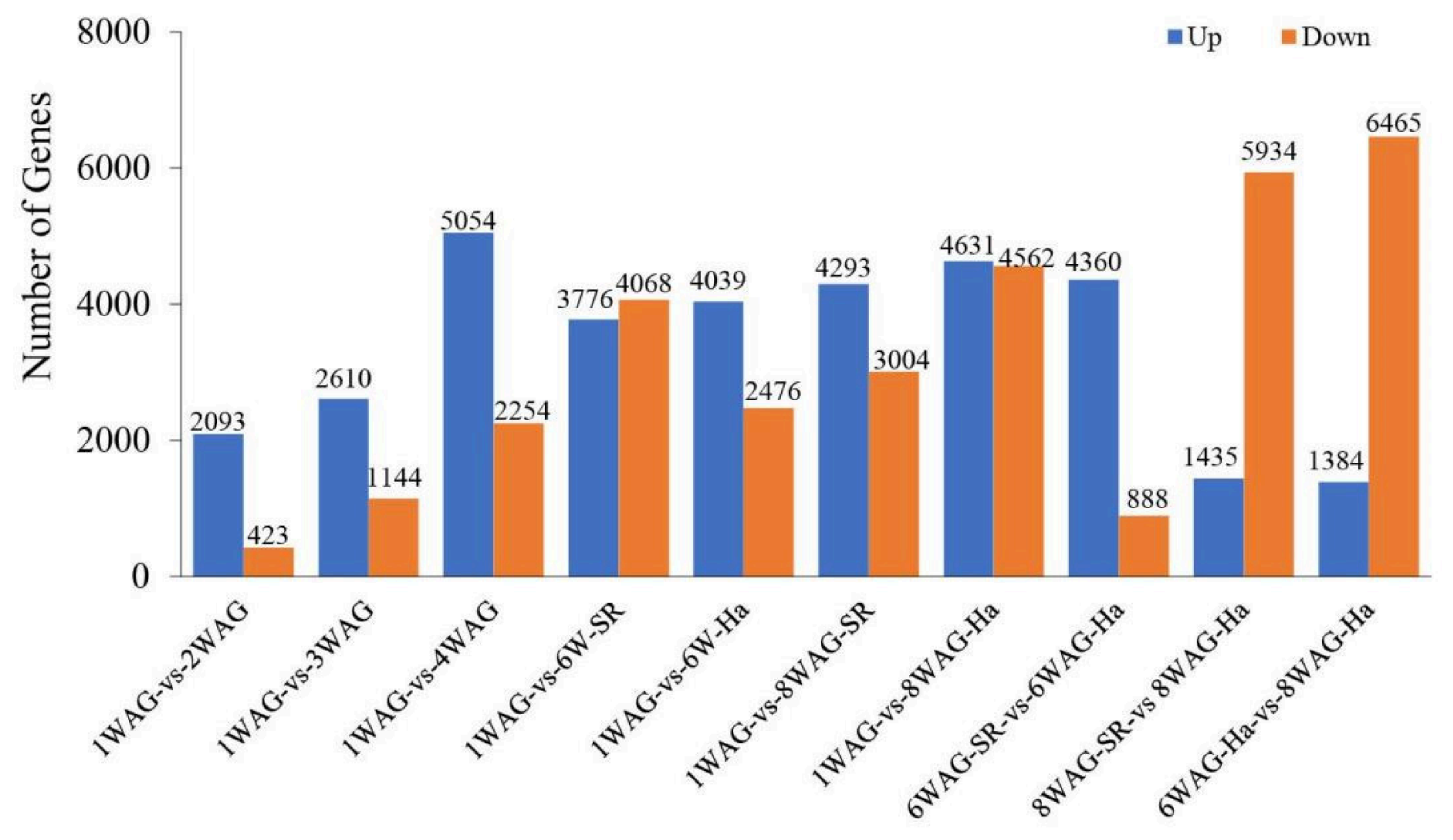
DEGs across developmental-stage comparisons of coconut zygotic embryos. Numbers of upregulated (blue) and downregulated (orange) genes identified in pairwise comparisons among coconut zygotic embryo developmental stages. Comparisons include early post-germination stages (1–4 WAG), shoot–root (SR) and haustorium (Ha) tissues at 6 and 8 WAG, and tissue-specific transitions between developmental stages. Values above the bars indicate the number of DEGs in each comparison. The results demonstrate extensive transcriptional reprogramming during embryo development, with particularly pronounced changes associated with haustorium maturation at 8 WAG. WAG, weeks after germination; SR, shoot–root tissue; Ha, haustorium.

Differential expression analysis revealed substantial transcriptional changes throughout coconut zygotic embryo development. Across the ten pairwise comparisons, the number of DEGs varied considerably in both magnitude and direction (Figure 1; Table S1). In comparisons with the early 1 WAG stage, the number of upregulated genes increased from 2093 genes at 2 WAG to 5054 genes at 4 WAG, while the number of downregulated genes increased from 423 to 2254 genes, respectively. At 6 WAG, 3776 and 4068 genes were up- and downregulated in the 1 WAG vs. 6 WAG-SR comparison, whereas 4039 and 2476 genes were differentially expressed in the 1 WAG vs. 6 WAG-Ha comparison.

At later developmental stages, the transcriptional differences became more pronounced. The comparison between 1 WAG and 8 WAG-SR identified 4293 upregulated and 3004 downregulated genes, while the 1 WAG vs. 8 WAG-Ha comparison showed 4631 upregulated and 4562 downregulated genes. Similarly, comparison between 6 WAG-SR and 6 WAG-Ha revealed 4360 upregulated and 888 downregulated genes, indicating substantial transcriptional divergence between the two tissues at 6 WAG.

The strongest shift toward downregulation was observed in comparisons involving 8 WAG tissues. The comparison between 8 WAG-SR and 8 WAG-Ha identified 1435 upregulated and 5934 downregulated genes. Similarly, the comparison between 6 WAG-Ha and 8 WAG-Ha revealed 1384 upregulated and 6465 downregulated genes, representing the largest number of downregulated genes among all comparisons. Overall, these results demonstrate extensive and stage-dependent transcriptional reprogramming during post-germination development, with particularly pronounced changes associated with haustorium maturation at 8 WAG.

### 2.2. Pearson Correlation Analysis of Transcriptomic Profiles Across Developmental Stages and Tissues

**Figure 2:**
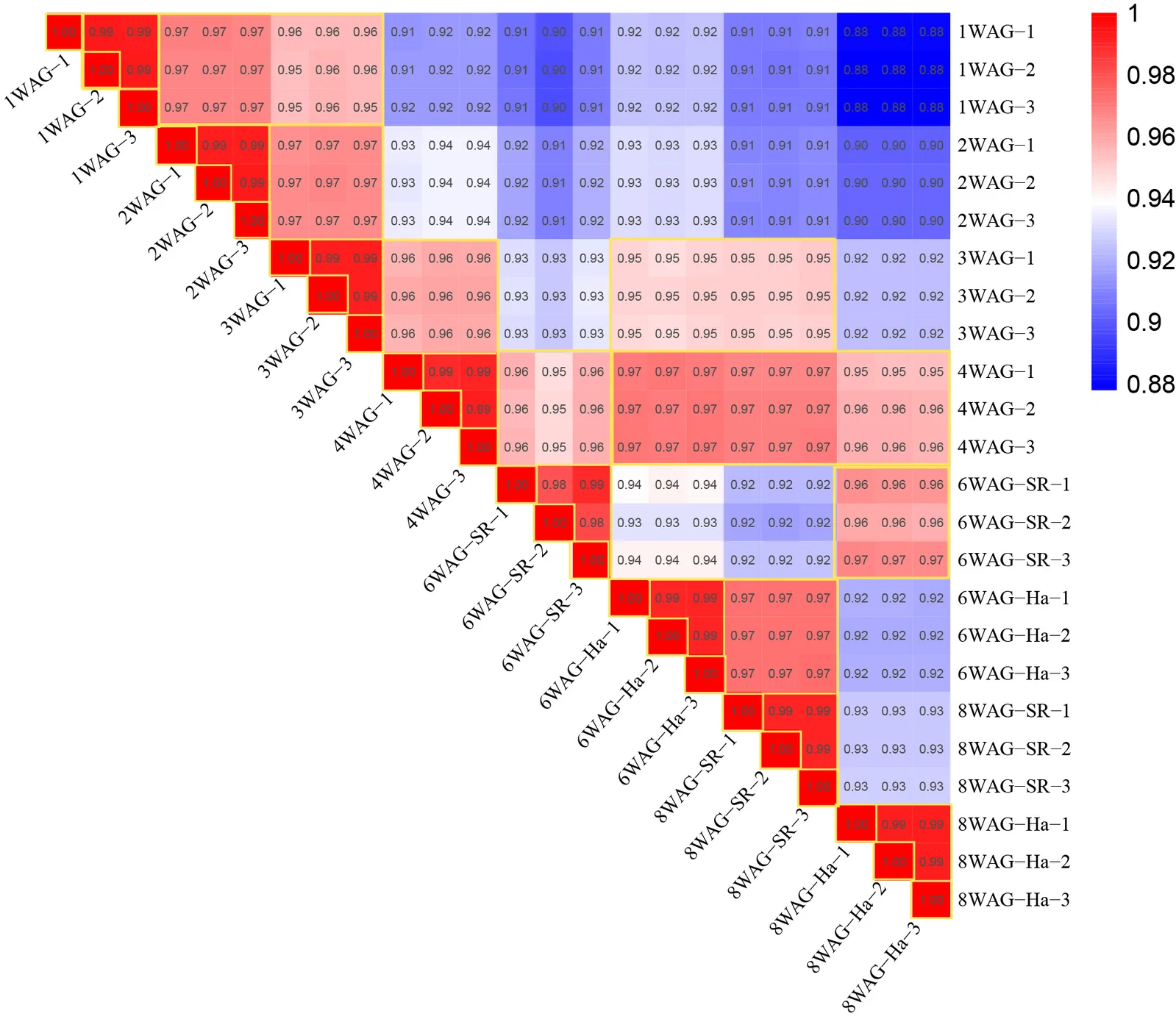
Pearson correlation heatmap showing relationships among transcriptomic samples during haustorium development. The heatmap demonstrates strong correlations among biological replicates, with correlation coefficients ranging from 0.88 to 1.00. Samples from the same developmental stage and tissue cluster together, indicating high reproducibility. Developmental progression from 1 WAG to 8 WAG results in increasing transcriptional divergence, particularly in 8 WAG haustorium samples, reflecting tissue-specific molecular changes during haustorium maturation.

Pearson correlation analysis was performed to evaluate the similarity and reproducibility among transcriptomic samples collected at different developmental stages (1, 2, 3, 4, 6, and 8 WAG) and tissues (shoot/root, SR; haustorium, Ha). Overall, all biological replicates showed highly consistent expression patterns, confirming the reliability of the transcriptomic datasets. Across developmental stages, transcriptomic profiles displayed a gradual decrease in similarity as developmental distance increased. Early-stage samples (1–3 WAG) were highly correlated with each other (r = 0.95–0.99), suggesting similar molecular characteristics during early development. The 4 WAG samples showed strong correlations with earlier developmental stages (r = 0.91–0.96) and later stages (r = 0.95–0.97), indicating a transitional transcriptional state between early and late developmental phases. The lowest correlations were observed between early 1 WAG samples and late 8 WAG-Ha samples (r = 0.88), suggesting progressive transcriptional divergence associated with haustorium maturation (Figure 2).

### 2.3. Common DEGs Across Developmental Stages

**Figure 3:**
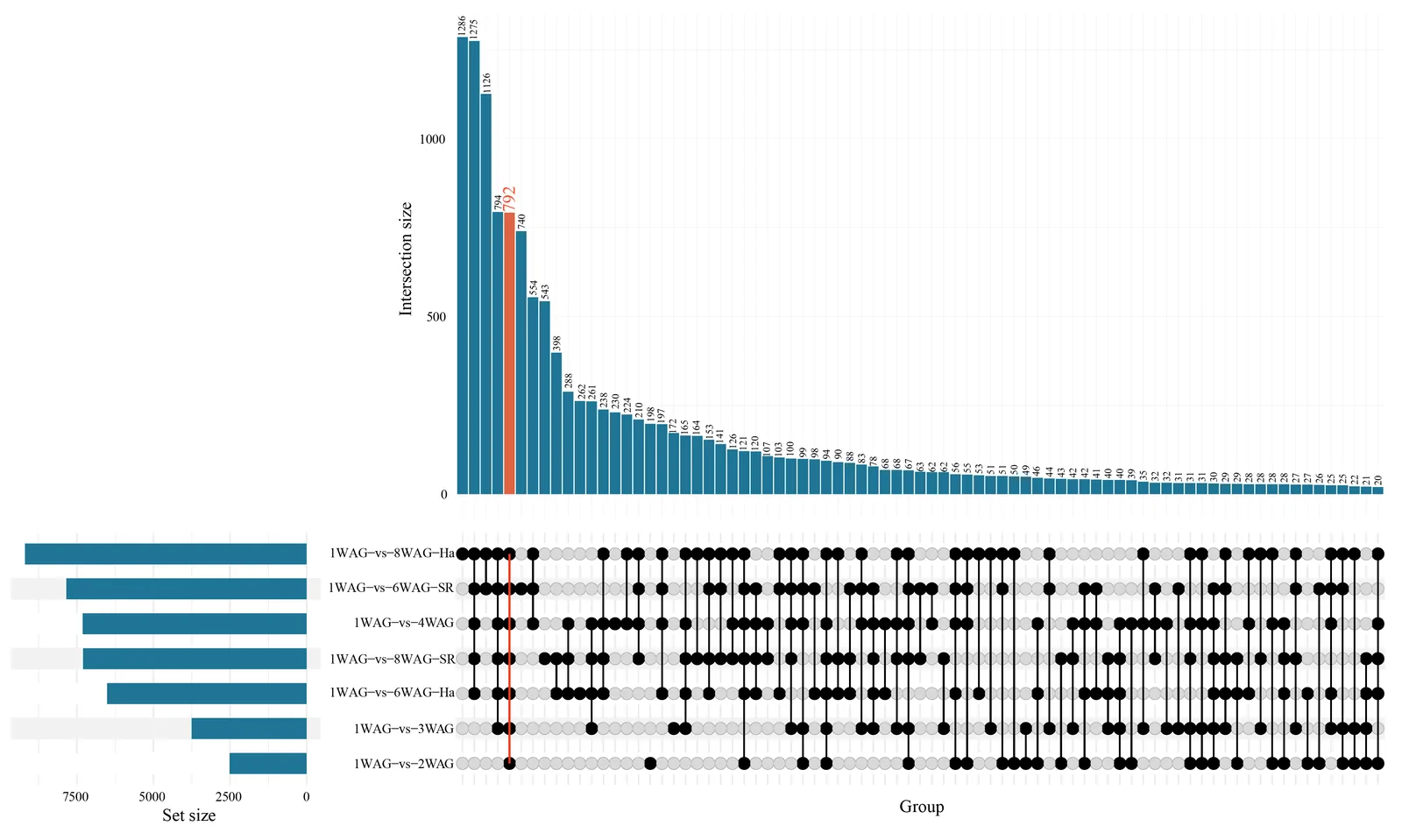
UpSet plot analysis of DEGs during coconut zygotic embryo development. UpSet plot showing the intersections of DEGs identified from pairwise comparisons between 1 WAG and subsequent developmental stages. The upper bar plot represents the number of DEGs in each intersection, while the lower matrix indicates the combinations of comparisons contributing to each intersection. A total of 792 DEGs were commonly identified across all seven developmental comparisons, including 1WAG vs. 2WAG, 3WAG, 4WAG, 6WAG-Ha, 6WAG-SR, 8WAG-Ha, and 8WAG-SR (highlighted in red). Horizontal bars on the left indicate the total number of DEGs detected in each comparison. These 792 shared DEGs represent a core set of genes consistently differentially expressed throughout post-germination development and were selected for subsequent clustering and functional enrichment analyses.

To identify core transcriptional changes associated with coconut zygotic embryo development after germination, an UpSet plot analysis was performed using DEGs identified from pairwise comparisons between 1 WAG and subsequent developmental stages. A total of 792 DEGs were shared across all seven comparisons, including 1WAG vs. 2WAG, 3WAG, 4WAG, 6WAG-Ha, 6WAG-SR, 8WAG-SR, and 8WAG-Ha (Figure 3; Table S2). Thus, these 792 genes represented a common developmental DEG set that remained differentially expressed relative to the 1 WAG stage throughout post-germination development.

The presence of this substantial shared DEG population indicates that a core transcriptional program is established early after germination and maintained or progressively reinforced during subsequent embryo development and haustorium differentiation. In contrast to stage-specific DEGs, the 792 common genes likely represent fundamental molecular changes associated with the transition from the early post-germination state toward later developmental stages. This conserved DEG set was therefore used for subsequent clustering and functional enrichment analyses to identify major biological processes underlying coconut zygotic embryo development.

### 2.4. Core Developmental DEGs Analysis

**Figure 4:**
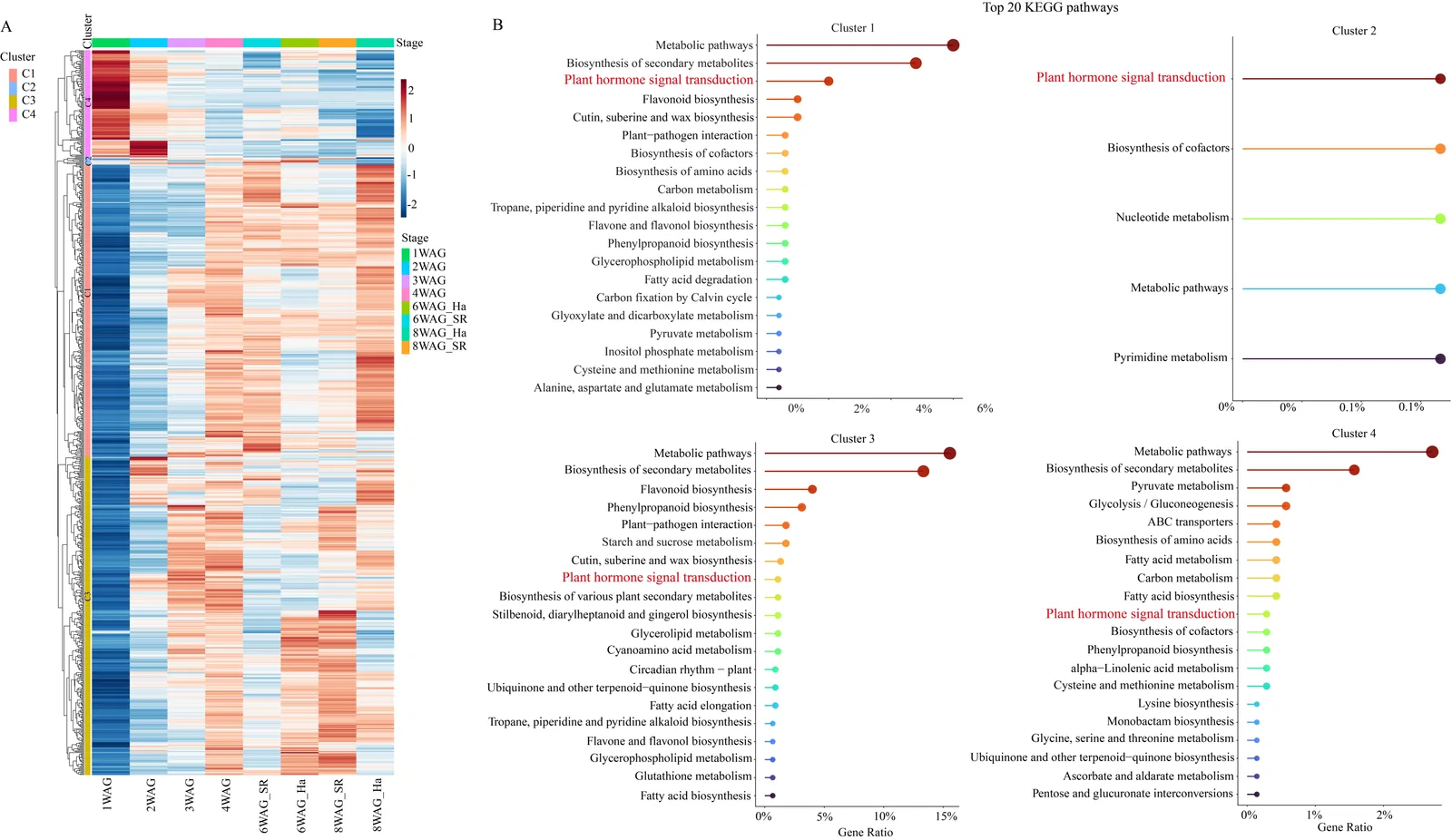
Expression profiles and KEGG pathway enrichment analysis across developmental stages. (**A**) Hierarchical clustering heatmap showing the expression patterns of DEGs during coconut zygotic embryo development (1WAG, 2WAG, 3WAG, 4WAG, 6WAG_SR, 6WAG_Ha, 8WAG_SR, and 8WAG_Ha). The expression level of each gene is scaled by Z-score, ranging from low expression (blue) to high expression (red). The color bar on the left indicates four distinct clusters (C1-C4). (**B**) Top 20 enriched KEGG pathways for gene Clusters 1 to 4. The horizontal axis indicates the gene ratio for each pathway. Key pathways shared or highlighted in red represent central regulatory mechanisms, such as plant hormone signal transduction.

We identified a set of core developmental DEGs shared across all seven developmental comparisons (1WAG vs 2WAG, 1WAG vs 3WAG, 1WAG vs 4WAG, 1WAG vs 6WAG-SR, 1WAG vs 6WAG-Ha, 1WAG vs 8WAG-SR, and 1WAG vs 8WAG-Ha). These core DEGs were subsequently analyzed to reveal transcriptional patterns and biological processes associated with coconut zygotic embryo development. Hierarchical clustering classified these genes into four major expression clusters (Cluster 1–4), each representing distinct developmental programs (Figure 4A; Table S3). KEGG pathway enrichment analysis revealed that each cluster was associated with specific biological processes, indicating functional specialization during embryo development (Figure 4B).

Cluster1 (C1) genes demonstrated low initial expression that steadily escalated through post-germination, peaking sharply in mature haustorial tissue (8WAG_Ha) (Figure 4A). KEGG pathways were heavily enriched in plant hormone signal transduction, Flavonoid biosynthesis, Cutin, suberine and wax biosynthesis, and plant–pathogen interaction. This late-stage haustorium-enriched profile highlights protective barrier formation and defense priming during final nutrient absorption stages (Figure 4B).

Cluster2 (C2) displayed a distinct developmental expression pattern characterized by activation mainly during early-to-middle developmental stages, particularly from 2–4 WAG, with several genes maintaining elevated expression during 6WAG_Ha. Compared with other clusters, C2 showed relatively lower overall expression intensity in the heatmap (Figure 4A). KEGG enrichment revealed that C2 genes were mainly associated with plant hormone signal transduction (Figure 4B), suggesting that C2 genes may function downstream of developmental hormone networks to regulate embryo growth and tissue differentiation.

Cluster3 (C3) genes exhibited increased expression mainly during middle-to-late developmental stages, particularly from 3–8 WAG, suggesting their involvement in embryo maturation and tissue specialization (Figure 4A). KEGG analysis demonstrated that C3 was strongly enriched in flavonoid biosynthesis, and phenylpropanoid biosynthesis (Figure 4B). Additional enriched pathways included starch and sucrose metabolism, and plant hormone signal transduction. The strong enrichment of phenylpropanoid and flavonoid pathways indicates increased production of specialized metabolites during late embryo development, potentially contributing to protection against environmental stresses and tissue maturation. The enrichment of carbohydrate metabolism suggests enhanced metabolic activity to support embryo expansion and developmental progression.

Genes in cluster4 (C4) exhibited high expression specifically at 1 week after germination (1WAG) and were rapidly downregulated in subsequent stages (Figure 4A). KEGG enrichment highlighted central energy-yielding processes, including Pyruvate metabolism, Glycolysis/Gluconeogenesis, ABC transporters, Biosynthesis of amino acids and Fatty acid metabolism (Figure 4B). This early-burst profile reflects initial seed reserve breakdown and transport needed to kickstart embryonic growth.

### 2.5. TF Analysis

**Figure 5:**
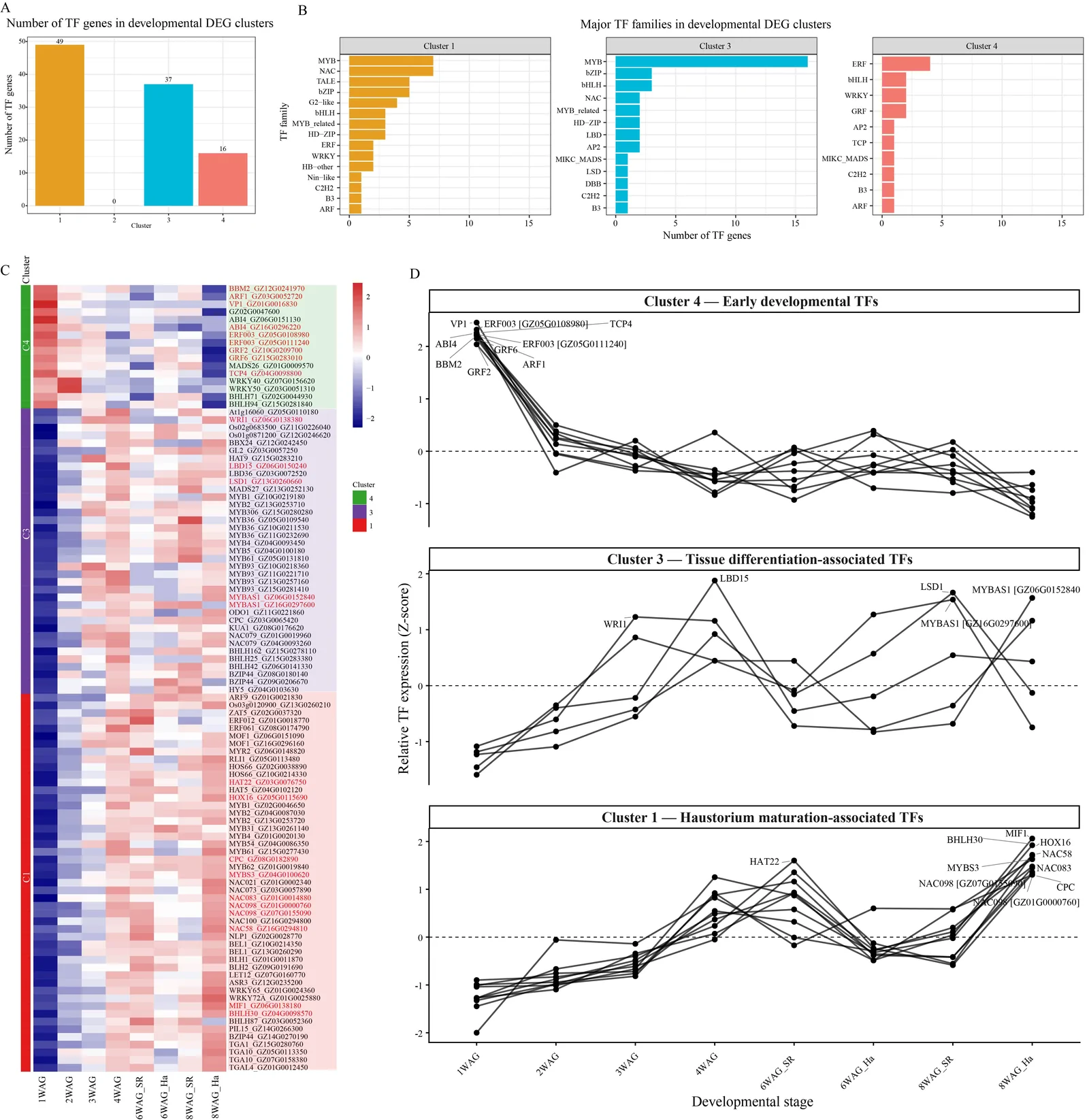
Distribution of transcription factor (TF) genes among developmental DEG clusters. (**A**) Number of annotated TF genes in each developmental DEG cluster. Cluster 1 contained the highest number of TF genes (49), followed by Cluster 3 (37) and Cluster 4 (16), whereas Cluster 2 contained no annotated TF genes. (**B**) Top transcription factor families identified in Clusters 1, 3, and 4. Cluster 1 was enriched in MYB, NAC, TALE, and bZIP transcription factors; Cluster 3 was dominated by MYB transcription factors; and Cluster 4 was characterized by ERF, bHLH, WRKY, and GRF families, indicating distinct transcriptional regulatory programs underlying coconut zygotic embryo development. (**C**) Developmental expression patterns of transcription factors (TFs) during coconut zygotic embryo germination and haustorium development. The heatmap shows standardized expression profiles of TFs across eight developmental and tissue conditions: 1WAG, 2WAG, 3WAG, 4WAG, 6WAG_SR, 6WAG_Ha, 8WAG_SR, and 8WAG_Ha. Expression values were transformed to gene-wise Z-scores across developmental stages, with red indicating relatively higher expression and blue indicating relatively lower expression than the gene-specific mean. TFs were grouped into three developmental expression clusters: Cluster 4 (green), early developmental TFs; Cluster 3 (purple), tissue differentiation-associated TFs; and Cluster 1 (red), haustorium maturation-associated TFs. Gene labels indicate the TF symbol followed by the corresponding coconut gene ID. The distinct expression patterns among clusters indicate dynamic and stage- and tissue-dependent regulation of TFs during embryo germination, tissue differentiation, and haustorium maturation. (**D**) Developmental expression trajectories of key transcription factors during coconut zygotic embryo germination and haustorium development. Gene-wise Z-scores were calculated from TPM expression values across eight developmental/tissue conditions (1WAG, 2WAG, 3WAG, 4WAG, 6WAG_SR, 6WAG_Ha, 8WAG_SR, and 8WAG_Ha). Individual lines represent TF expression trajectories, and TF symbols are positioned at the developmental stage exhibiting the maximum Z-score. TFs are grouped into Cluster 4 (early developmental TFs), Cluster 3 (tissue differentiation-associated TFs), and Cluster 1 (haustorium maturation-associated TFs). Positive and negative Z-scores indicate expression above and below the TF-specific mean, respectively.

A total of 102 transcription factor (TF) genes belonging to multiple TF families were identified among the 792-core developmental DEGs. These TFs were unevenly distributed across the four expression clusters. Cluster 1 contained the largest number of TF genes (49), followed by Cluster 3 (37) and Cluster 4 (16), whereas Cluster 2 contained no annotated TF genes, indicating that this small cluster is unlikely to be regulated primarily by transcriptional regulators (Figure 5A; Table S4).

Analysis of the major TF families revealed clear differences among clusters. In Cluster 1, the most abundant TF family was MYB (7 genes), followed by NAC (7 genes), TALE (5 genes), bZIP (5 genes), G2-like (4 genes) and bHLH (3 genes). Other TF families, including MYB-related, HD-ZIP, ERF, WRKY, HB-other, Nin-like, C2H2, B3, and ARF, were represented by one to three genes. The enrichment of MYB, NAC, TALE, and bZIP families suggests that Cluster 1 is associated with extensive transcriptional regulation involved in developmental reprogramming, meristem establishment, and hormone-mediated signaling (Figure 5B; Table S4).

In Cluster 3, the MYB family was overwhelmingly dominant (16 genes), accounting for nearly half of all TFs in this cluster. Additional TF families included bZIP (3 genes), bHLH (3 genes), NAC (2 genes), MYB-related (2 genes), HD-ZIP (2 genes), LBD (2 genes) and AP2 (2 genes), while MIKC_MADS, LSD, DBB, C2H2, and B3 were each represented by a single gene. The predominance of MYB transcription factors suggests an important role in regulating developmental differentiation, secondary metabolism, and vascular tissue formation during embryo development (Figure 5B; Table S4).

In Cluster 4, the ERF family was the largest TF group (4 genes), followed by bHLH (2 genes), WRKY (2 genes) and GRF (2 genes). Several additional TF families, including AP2, TCP, MIKC_MADS, C2H2, B3, and ARF, were each represented by a single gene. The enrichment of ERF and WRKY transcription factors indicate that genes in this cluster are likely involved in ethylene-responsive signaling, stress adaptation, and late developmental processes, whereas GRF and TCP may contribute to organ growth and cell proliferation (Figure 5B; Table S4).

Overall, the TF family analysis demonstrates that each developmental DEG cluster possesses a distinct regulatory signature. Clusters 1 and 3 were dominated by MYB- and NAC-related transcriptional regulators, suggesting active control of developmental and differentiation processes, whereas Cluster 4 was characterized by ERF- and WRKY-mediated regulatory pathways, reflecting functions associated with tissue maturation and environmental response. The absence of annotated TF genes in Cluster 2 suggests that this cluster mainly comprises structural or metabolic genes rather than transcriptional regulators.

Cluster 1: Haustorium maturation-associated TFs

Cluster 1 TFs exhibited diverse expression trajectories across developmental stages and tissues, although most showed relatively low transcript abundance during early germination. Of the 49 TF genes, 33 reached their highest mean expression at 8WAG-Ha, indicating that a substantial proportion of Cluster 1 TFs were associated with late-stage haustorium development. However, the remaining TFs showed peak expression at other developmental stages, including 3WAG, 4WAG, 6WAG-SR, 6WAG-Ha, and 8WAG-SR (Figure 5C; Table S4). These differences indicate that Cluster 1 contains TFs with distinct developmental and tissue-associated expression patterns, rather than a uniformly increasing expression trajectory. Nevertheless, the predominance of TFs with elevated expression in mature haustorial tissue supports an association between Cluster 1 and haustorium maturation. This expression pattern is consistent with the biological processes involved in haustorial maturation, including intrusive cell differentiation, vascular bridge formation, secondary cell wall biosynthesis, and nutrient transfer between the haustorium and the endosperm [11].

The Cluster 1 TF repertoire encompassed 17 TF families. Three candidate regulatory TFs were prioritized based on their high expression levels, and substantial fold induction from 1WAG to 8WAG_Ha. MIF1 was the most highly expressed TF in Cluster 1 at 8WAG_Ha, with a mean expression level of 427.74, representing a 16-fold increase compared with 1WAG and 2.6-fold higher expression in the haustorium than in the corresponding shoot/root tissue at 8WAG. Mini Zinc Finger (MIF) proteins belong to the plant-specific ZF-HD superfamily, and have been implicated in the integration of hormonal signals, including auxin, abscisic acid (ABA), and gibberellins, in the regulation of cell elongation, tissue morphogenesis, and stress responses [12]. These reported functions suggest that MIF1 may contribute to the developmental processes associated with coconut haustorium differentiation. ZF-HD/MIF TFs have been implicated in the regulation of cell fate transitions during specialized lateral organ formation, suggesting a potential role for MIF1 in late-stage haustorium differentiation. HOX16 was the second-most highly expressed Cluster 1 TF at 8WAG-Ha, with a mean expression level of 315.99, representing a 13-fold increase compared with 1WAG and 1.9-fold higher expression in the haustorium than in the corresponding shoot/root tissue. HD-ZIP TFs are known to regulate vascular patterning, epidermal cell identity, and apical-basal tissue polarity in developing plant organs [13]. Consistent with these established developmental functions, the late-stage haustorium-biased expression of HOX16 suggests a possible involvement in tissue differentiation and haustorium development during coconut embryo germination. Two NAC098 paralogs exhibited among the strongest haustorium specificity in Cluster 1 (4.2× and 3.8× higher expression in 8WAG_Ha vs. 8WAG_SR), with near-undetectable expression at 1WAG and explosive late-stage induction (273-fold and infinite fold change from zero baseline). NAC-domain transcription factors are important regulators of xylem differentiation, secondary cell wall deposition, and developmentally regulated programmed cell death (PCD) in plant vascular tissues [14]. Additional candidate NAC TFs included NAC58, which showed 7.5-fold higher expression in the haustorium than in the corresponding shoot/root tissue and a 266-fold increase from 1WAG, and NAC083, which exhibited a mean expression level of 137.21 at 8WAG-Ha. The expression patterns of these TFs suggest their potential involvement in vascular tissue differentiation, cell wall remodeling, and tissue development during late-stage coconut haustorium maturation (Figure 5D).

Other notable Cluster 1 TFs with large fold induction and haustorial enrichment included MYBS3 (MYB-related, 156-fold induction), HAT22 (HD-ZIP, 3.6× haustorium specificity), BHLH30 (bHLH, 99-fold upregulation), and CPC (MYB-related, 179.37 expression at 8WAG_Ha). Collectively, the Cluster 1 TF expression profiles suggest the involvement of these transcription factors in late-stage coconut haustorium development, particularly in tissue differentiation, vascular development, and processes associated with nutrient absorption and transport.

Cluster 3: Tissue differentiation-associated TFs

Cluster 3 TFs were weakly expressed at 1–2 WAG, with robust, sustained transcriptional activation from 3WAG through all 6 and 8WAG tissue timepoints (Figure 5C; Table S4), consistent with a regulatory role in embryo maturation, storage compound biosynthesis, and general tissue specialization during mid-to-late post-germination development. Unlike Cluster 1, Cluster 3 TFs exhibited moderate expression in both seedling root and haustorial tissue, indicating pleiotropic functions in multiple maturing organ systems rather than exclusive haustorial specialization. Cluster 3 contained TFs from 13 families, with three top candidates selected for extreme expression magnitude, canonical roles in seed maturation, and consistent mid-to-late-stage induction. LSD1 was the most highly expressed TF in Cluster 3, peaking at 309.15 in 8WAG_SR and 230.67 at 4WAG, with 17-fold induction from 1WAG. LESION SIMULATING DISEASE 1 (LSD1) is a conserved negative regulator of oxidative stress-triggered PCD, functioning as a molecular hub balancing developmental cell death, basal defense signaling, and tissue homeostasis during organ maturation [15]. Its extreme mid-to-late expression suggests LSD1 coordinates redox homeostasis and cell survival in maturing embryo and root tissues to support sustained organ growth. WRI1 transcript levels increased sharply from 3 WAG onward, with elevated expression in both maturing seedlings and haustoria at 8WAG. WRINKLED1 is the master AP2 TF governing seed embryo maturation, directly activating the complete glycolysis and fatty acid triacylglycerol biosynthesis pathways required for storage lipid accumulation in developing seeds [16]. WRI1’s mid-to-late upregulation aligns perfectly with the Cluster 3 functional signature of embryo maturation and storage compound deposition, marking it a core regulator of late-stage seed developmental programs. Two MYBAS1 paralogs showed abundant mid-to-late expression, peaking at 209.62 in 8WAG_Ha and 35.36 in 6WAG_Ha, with 2.5–4.9-fold induction from early germination. MYB-type TFs including MYBAS1 are central activators of the phenylpropanoid and anthocyanin biosynthetic pathways, which accumulate secondary metabolites for structural reinforcement, stress tolerance, and pigmentation during tissue maturation. Additional functionally relevant Cluster 3 TFs included the LBD family member LBD15, which showed progressive upregulation from 3 WAG onward. LBD15 was weakly expressed during the early post-germination stages (1–2WAG) and reached its highest expression level (12.31) at 4WAG, consistent with the mid-to-late developmental program represented by Cluster 3 (Figure 5D). LBD transcription factors govern lateral organ boundary formation, embryonic tissue patterning, and vascular differentiation during organ maturation, making them secondary candidate regulators coordinating tissue specialization across seedling root and haustorial tissues [17,18].

Other notable mid-late-stage regulators included HY5 and BZIP44 (bZIP light-signaling regulators), NAC079 paralogs (strong 18–22-fold mid-stage induction), MADS27 (MIKC_MADS developmental patterning TF), and ODO1 (MYB phenylpropanoid regulator). This TF cohort establishes a shared mid-to-late developmental regulatory network governing lipid storage, secondary metabolism, redox homeostasis, and tissue differentiation across seedling and haustorial organs.

Cluster 4: Early developmental TFs

Cluster 4 TFs exhibited a sharply distinct expression signature: maximal transcript abundance exclusively at 1WAG, followed by rapid, progressive transcriptional repression across all subsequent developmental timepoints (2–8WAG) (Figure 5C; Table S4). This pattern identifies a TF module dedicated to regulating the immediate post-germination transition from dormant seed to proliferative seedling, coordinating cell proliferation, hormone signaling, and early embryogenic competence before tissue and organ differentiation programs dominate mid/late development. Cluster 4 TFs spanned 10 TF families, with three master early germination regulators prioritized for maximal 1WAG expression and steep post-1WAG downregulation.

GRF2 was the highest-expressed TF genome-wide at 1WAG (131.86), with a 3.2-fold higher abundance at 1WAG relative to all later stages. Growth-regulating factor (GRF) TFs are core positive regulators of cell cycle progression and cell proliferation in young, newly germinated tissues, driving hypocotyl, radicle, and apical meristem outgrowth immediately following radicle emergence. Its dramatic early peak identifies GRF2 as the primary TF orchestrating post-germination cell division and seedling establishment. ABI4 showed strong, specific 1WAG expression (54.12) followed by near-complete transcriptional silencing at later timepoints (5.9-fold higher at 1WAG vs. mature tissues). ABI4 is a canonical ABA-signaling TF that mediates the antagonistic ABA–gibberellin hormone crosstalk governing seed dormancy release and germination initiation; it represses GA biosynthetic genes and stabilizes ABA signaling to modulate the seed-to-seedling developmental switch [19,20]. Its exclusive early expression positions ABI4 as a critical checkpoint regulator of germination competence. BBM2 (BABY BOOM 2) peaked at 1WAG (9.99) with 3.8-fold early-stage enrichment (Figure 5D). AP2-type BBM TFs maintain embryonic cell identity and promote meristematic cell proliferation in newly germinated seedlings, sustaining embryogenic transcriptional programs before tissue differentiation pathways are activated at 2WAG and beyond. Other prominent Cluster 4 TFs included BHLH94 (100.46 expression at 1WAG), ERF003 paralogs (ethylene hormone signaling), VP1 (B3 seed maturation/germination repressor, 27.6× 1WAG specificity), TCP4 (cell cycle and organ boundary regulation) [21], and ARF1 (auxin response). Collectively, Cluster 4 TFs constitute a transient early transcriptional cascade governing hormone balance, cell proliferation, and embryonic identity during the critical 1-week post-germination developmental window.

### 2.6. Co-Expression Network of Early Zygotic Embryo Germination

**Figure 6:**
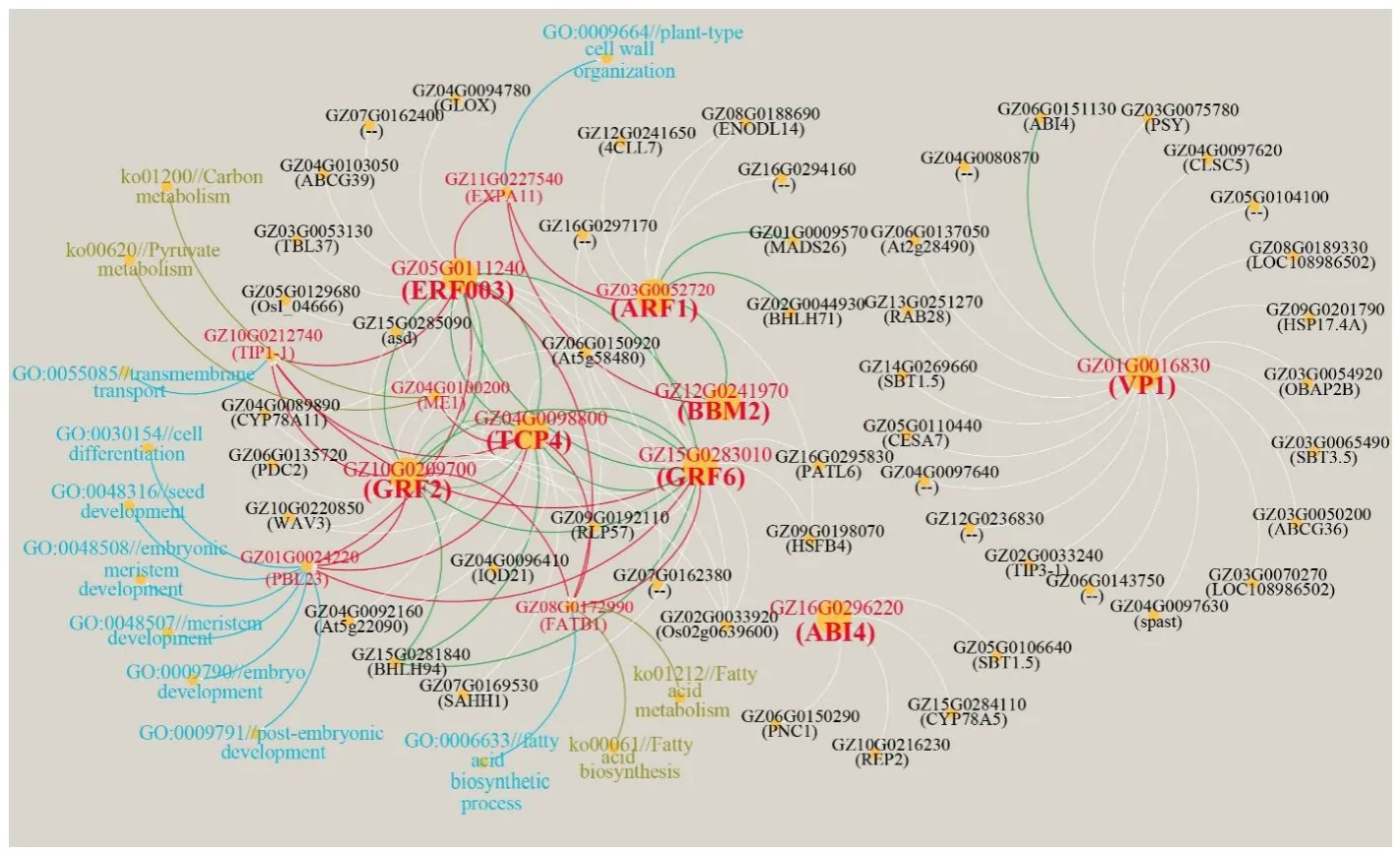
Gene co-expression network associated with early zygotic embryo germination (cluster 4). Nodes represent individual genes, and edges indicate significant positive |r| ≥ 0.90 correlations between gene pairs. Bold red labels represent focal TFs; regular red labels denote critical genes highly co-expressed with multiple focal TFs. Blue texts correspond to Gene Ontology (GO) biological process terms, and olive-green texts represent Kyoto Encyclopedia of Genes and Genomes (KEGG) enrichment terms for these critical genes, whose functions are closely associated with early zygotic embryo germination. Green edges stand for TF-to-TF correlation relationships, and red edges represent correlation between focal TFs and their co-expressed critical genes.

Cluster 4 TF–TF–gene network reveals a hierarchical regulatory program governing early coconut embryo development. VP1 and ABI4 showed co-expression patterns associated with ABA-related maturation processes, while the TCP4-ERF003-GRF2-GRF6-BHLH94 clique and the ARF1-0BBM2-MADS26-BHLH71 module comprised TFs potentially associated with cell proliferation, expansion, and tissue patterning. These TF modules converge on a relatively small set of effector genes-PBL23 (developmental patterning), EXPA11 (cell wall remodeling), FATB1/ME1 (carbon and lipid metabolism), and TIP1-1 (vacuolar transport)-whose coordinated expression underpins the transition from early embryogenesis to seed maturation (Figure 6). The network provides insights into potential co-expression relationships among multiple TF families and genes associated with developmental and metabolic processes during the early stages of coconut embryo formation.

### 2.7. Co-Expression Network of Haustorium Maturation

**Figure 7:**
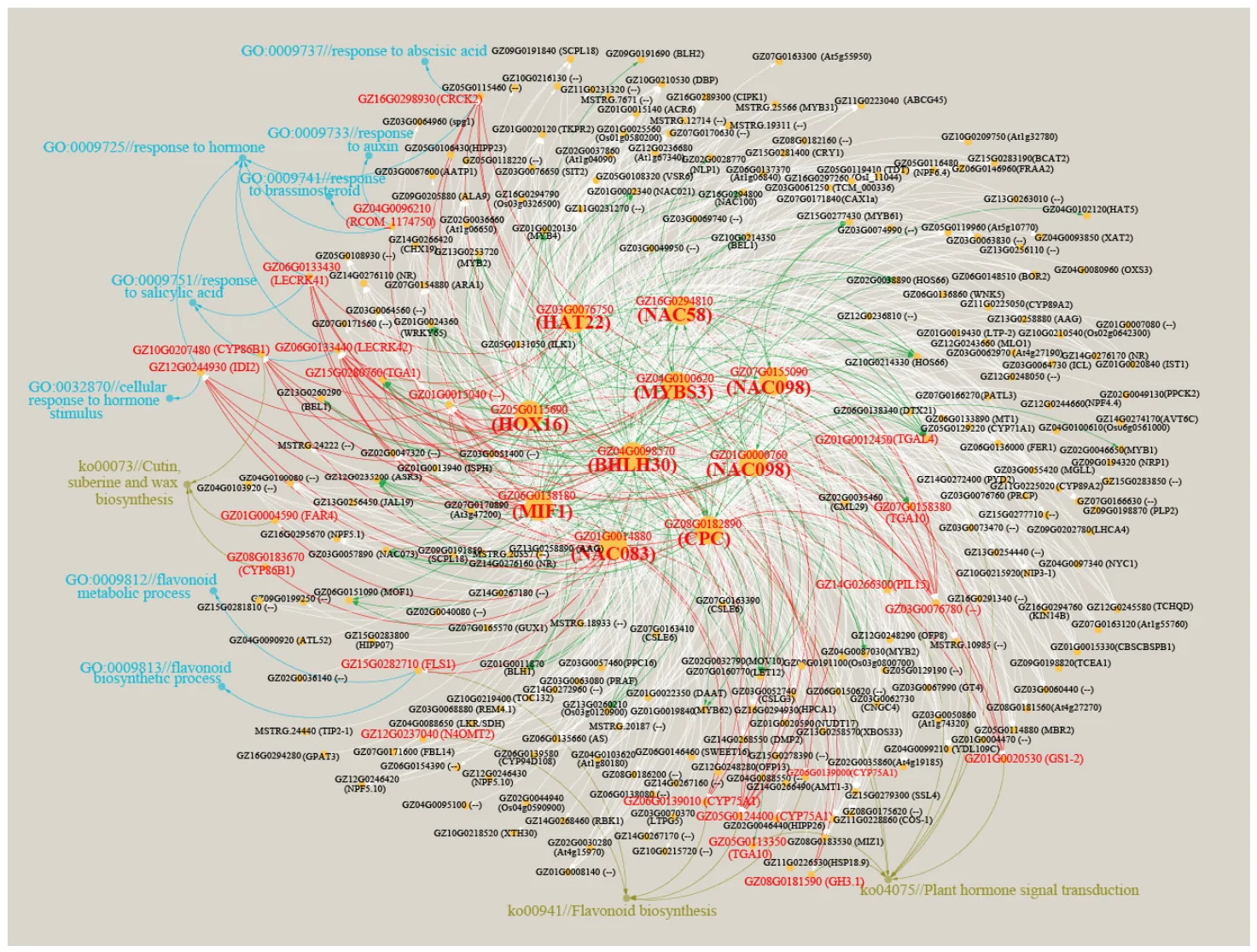
Gene co-expression network associated with haustorium maturation (cluster 1)**.** Nodes represent individual genes, and edges indicate significant positive |r| ≥ 0.90 correlations between gene pairs. Bold red labels represent focal TFs; regular red labels denote critical genes highly co-expressed with multiple focal TFs. Blue texts correspond to Gene Ontology (GO) biological process terms, and olive-green texts represent Kyoto Encyclopedia of Genes and Genomes (KEGG) enrichment terms for these critical genes, whose functions are closely associated with early zygotic embryo germination. Green edges stand for TF-to-TF correlation relationships, and red edges represent correlation between focal TFs and their co-expressed critical genes.

To investigate the regulatory program associated with Cluster 1, which showed progressive upregulation during post-germination and was strongly associated with haustorium maturation, correlation analysis was performed between ten focal transcription factors (TFs) and other TFs and genes (Figure 7). The resulting network revealed extensive co-expression relationships, with *MIF1* (GZ06G0138180), *HOX16* (GZ05G0115690), and *CPC* (GZ08G0182890) showing the highest connectivity (degree = 154, 153, and 116, respectively), suggesting that these TFs occupy central positions within the Cluster 1 regulatory network.

The network contained extensive TF–TF associations, with 480 correlations involving multiple TF families, including *NAC*, *MYB*, *bHLH*, *TGA*, *ERF*, *ARF*, *WRKY*, *BEL*, and *bZIP*. Strong associations among focal TFs, including *MIF1-HAT22*, *NAC083-NAC098*, *NAC098-CPC*, and *MYBS3-NAC58*, further supported coordinated transcriptional regulation during haustorium maturation.

Functional annotation indicated that the network was strongly associated with hormone-responsive processes, including responses to abscisic acid, auxin, salicylic acid, and brassinosteroid. Consistently, KEGG analysis identified the plant hormone signal transduction pathway (ko04075), with genes such as *TGAL4*, *TGA1*, *PIL15*, *TGA10*, *GS1-2*, and *GH3.1* represented in the network. In addition, flavonoid biosynthesis (ko00941) was represented by genes including *CYP75A1*, *N4OMT2*, and *FLS1*, while *CYP86B1* and *FAR4* were associated with cutin, suberine, and wax biosynthesis (ko00073). Together, these results indicate that the Cluster 1 regulatory network integrates TF-mediated transcriptional coordination with hormone signaling, secondary metabolism, and protective barrier-related processes during haustorium maturation.

### 2.8. Developmental Changes in Hormone Profiles

**Figure 8:**
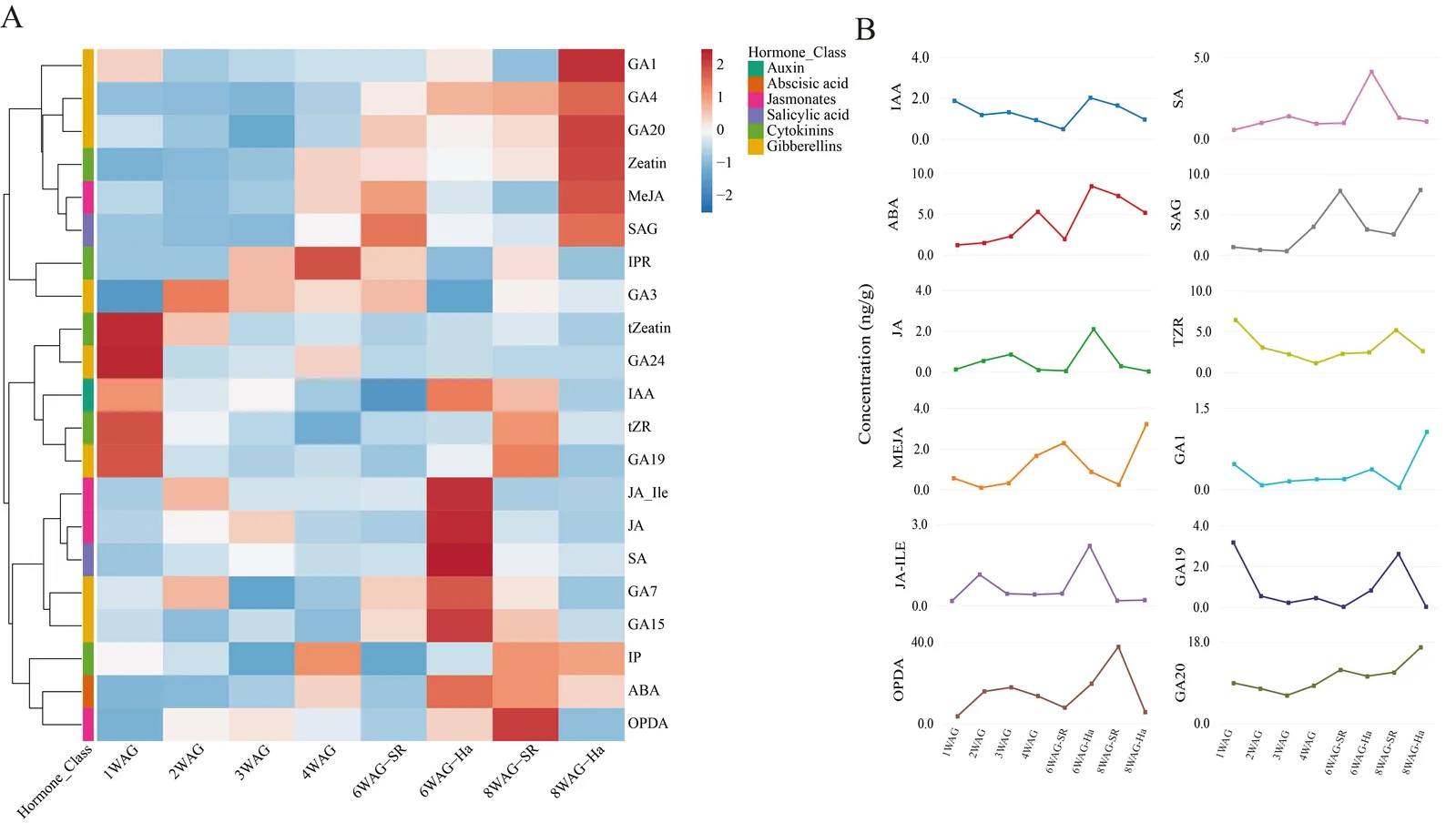
Dynamics of phytohormones during coconut zygotic embryo germination. (**A**) Heatmap showing the relative abundance of phytohormones across developmental stages from 1 to 8 weeks after germination (WAG). Hormone concentrations were normalized and represented as Z-scores, with red indicating relatively higher and blue indicating relatively lower abundance. Hormones were hierarchically clustered according to their abundance patterns across developmental stages. The colored annotation bar indicates the corresponding hormone class, including auxins, abscisic acid, jasmonates, salicylic acid, cytokinins, and gibberellins. (**B**) Temporal profiles of selected phytohormones showing representative changes in hormone concentrations across developmental stages. Hormones with very low or near-detection-limit concentrations were excluded from the line plots for clarity and to facilitate visualization of the major hormone dynamics. Hormone concentrations are expressed as ng/g fresh weight. Samples included 1WAG, 2WAG, 3WAG, 4WAG, 6WAG-SR, 6WAG-Ha, 8WAG-SR, and 8WAG-Ha, where SR represents shoot/root tissues and Ha represents haustorium tissue. Each line represents the developmental trajectory of an individual selected hormone.

Phytohormone profiling revealed distinct developmental changes in hormone abundance during coconut zygotic embryo germination and haustorium development (Table S5). Hierarchical clustering separated hormones according to their temporal profiles, indicating coordinated and hormone-specific patterns (Figure 8A).

Auxin (IAA) generally declined during early development but showed a transient increase in 6-WAG-Ha tissue, suggesting localized auxin accumulation during early haustorium differentiation. ABA showed a strong developmental increase, reaching its highest level at 6-WAG-Ha and remaining relatively abundant at 8WAG.

Jasmonate-related metabolites displayed distinct dynamics. JA and JA-Ile were strongly enriched in 6-WAG-Ha, whereas MeJA increased toward 8-WAG-Ha. OPDA showed a particularly pronounced accumulation, reaching its highest level in 8-WAG-SR. Similarly, SA increased strongly in 6-WAG-Ha, while its conjugated form SAG accumulated in 6-WAG-SR and 8-WAG-Ha, indicating developmental changes in SA metabolism.

Cytokinin metabolites generally showed higher abundance during early development, with tZR showing a later increase in 8-WAG-SR, whereas tZeatin progressively declined toward the later stages. Gibberellins exhibited diverse developmental patterns, with GA20 increasing progressively and reaching its highest abundance in 8-WAG-Ha, while GA1 and GA4 also increased in later haustorial tissue. In contrast, GA19 showed an overall decline after the early stages, with a transient increase in 8-WAG-SR (Figure 8A,B).

## 3. Discussion

The present study identified distinct transcriptional programs associated with early coconut zygotic embryo development, tissue differentiation, and haustorium maturation. By integrating differential expression analysis, TF co-expression networks, and phytohormone profiling, we identified candidate regulators and developmental pathways that complement previous findings on plant embryo development and hormone signaling. Unlike studies focused primarily on individual developmental stages, this analysis provides a time-resolved view of the molecular changes accompanying coconut embryo germination and haustorium development.

An important consideration in interpreting the present developmental transcriptomic profiles is the difference in sampled materials between early and later developmental stages. Whole zygotic embryos were analyzed at 1–4 WAG, whereas separate shoot/root (SR) and haustorium (Ha) tissues were collected at 6 and 8 WAG, following the complete outgrowth and morphological differentiation of these structures. Consequently, the observed transcriptional changes across the entire sampling period may reflect a combination of developmental progression and changes in tissue composition. In particular, differences between early whole-embryo samples and later SR or Ha samples should not be attributed exclusively to temporal regulation within the same tissue. Nevertheless, comparisons between SR and Ha at the same developmental stages provide additional insights into tissue-associated transcriptional differences during later development. Thus, the expression clusters identified in this study are best interpreted as developmental- and tissue-associated transcriptional patterns rather than strictly continuous temporal changes within a single tissue type. Future studies using consistently sampled tissues or spatially resolved transcriptomic approaches will help distinguish developmental regulation from tissue-specific expression more precisely.

With this sampling context in mind, the Cluster 4 co-expression network was associated with early embryo development and included TFs related to embryo maturation, hormonal responses, cell proliferation, and tissue patterning.

VP1 (GZ01G0016830), the highest-degree hub in this network, was co-expressed with RAB28, HSP17.4A, TIP3-1, and PSY. This pattern is consistent with the established role of B3-domain transcription factors in seed maturation and the regulation of germination [22,23]. VP1 was also connected with ABI4 (GZ06G0151130), a regulator of ABA-associated developmental processes suggesting an ABA-associated regulatory axis involved in maturation and dormancy [24,25]. Together, these associations suggest that VP1 and ABI4 may participate in a transcriptional module related to early developmental regulation and maturation-associated processes in coconut embryos.

A second prominent module contained TCP4, ERF003, GRF2, GRF6, and BHLH94, with 9 of the 10 possible pairwise co-expression edges. This module likely coordinates cell proliferation and expansion during early embryogenesis [26]. GRF proteins have been associated with cell proliferation and embryogenic development [8,9,27], while ERF and bHLH factors participate in diverse developmental and environmental responses [28,29,30]. These TFs were connected with genes including PBL23, FATB1, ME1, TIP1-1, which are associated with developmental processes, lipid and carbon metabolism, and cellular transport. In addition, ARF1 was connected with BBM2, MADS26, and BHLH71. Previous studies have established important roles for auxin response factors in embryonic patterning and root development [3,31,32], while MADS-box proteins participate in seed and reproductive development [10,33,34]. Similar interactions between transcriptional regulators and hormone signaling pathways have been reported during plant developmental transitions and embryogenic processes, including in coconut [35].

The observed co-expression relationships therefore identify candidate modules through which hormonal responses and developmental regulators may be coordinated during early coconut embryo development.

Cluster 3 showed an expression pattern associated with tissue differentiation, with 37 annotated TFs and a predominance of MYB-family members. This pattern distinguishes the differentiation-associated program from the early developmental program in Cluster 4 and the haustorium-associated program in Cluster 1. The results indicate that coconut embryo germination involves temporally distinct transcriptional responses rather than a single uniform developmental program. Further functional studies will be needed to establish the specific contributions of Cluster 3 TFs to shoot and root differentiation.

In contrast to the early expression pattern of Cluster 4, Cluster 1 showed a progressive upregulation during development and was strongly associated with haustorium maturation. MIF1, HOX16, and CPC had the highest connectivity in the Cluster 1 network, indicating that they are prominent candidate regulators within this co-expression module. Functional enrichment highlighted plant hormone signal transduction, flavonoid biosynthesis, and cutin, suberine, and wax biosynthesis. Genes including CYP75A1, N4OMT2, and FLS1 were associated with flavonoid biosynthesis, whereas CYP86B1 and FAR4 were associated with lipid-related protective barrier pathways. These results suggest that haustorium maturation involves coordinated changes in hormone-responsive transcription, secondary metabolism, and processes potentially related to surface protection. This interpretation is consistent with the specialized role of the coconut haustorium in supporting nutrient acquisition from the endosperm [1,2].

Phytohormone profiling revealed additional developmental differences. Auxin- and cytokinin-related metabolites were generally more abundant during early development, whereas ABA increased during development and reached a high level in 6WAG-Ha. These patterns are consistent with the established involvement of auxin in cell division and tissue differentiation and of ABA in seed maturation and developmental transitions [3,4,5,6,7]. In the related palm species Butia capitata, haustorium development was associated with increased IAA and gibberellin activity, changes in ABA, and alterations in cytokinin and brassinosteroid levels, indicating coordinated hormonal regulation of haustorium growth and reserve mobilization [36].

The elevated IAA, JA, JA-Ile, and SA levels in 6WAG-Ha also coincided with the enrichment of hormone-responsive and defense-associated processes in Cluster 1. JA and SA are known to participate in plant defense and stress responses [37,38]; therefore, their accumulation may reflect the physiological changes accompanying haustorium differentiation. The further increases in MeJA and SAG in 8WAG-Ha suggest continued changes in jasmonate and salicylic acid metabolism during later development. These relationships are consistent with coordinated hormonal and transcriptional changes, although the present data cannot establish direct causal links between hormone abundance and gene expression. Nevertheless, the phytohormone profiles should be interpreted descriptively because independent biological replicates were not included in this study. Therefore, further studies incorporating biological replication are needed to validate the observed developmental patterns and determine whether differences in hormone concentrations among developmental stages are statistically significant.

In addition to its theoretical significance in elucidating the molecular coordination of monocotyledonous zygotic embryo germination and haustorium specialization, this study provides actionable practical applications for palm biotechnology. Given that somatic embryogenesis and genetic transformation in coconut remain severely constrained by inefficient regeneration systems, the stage-specific transcription factor modules (such as the early embryogenic regulators BBM2 and GRF2 identified in Cluster 4) offer promising genetic targets. Manipulating these candidate regulators or optimizing culture media based on our phytohormone profiling data could significantly advance in vitro tissue culture, micropropagation, and molecular breeding programs in coconut and other related tropical palm crops.

Taken together, the results support a developmental model in which distinct transcriptional programs accompany early embryo development, tissue differentiation, and haustorium maturation. The Cluster 4 network identified candidate TF modules involving VP1, ABI4, ARF1, BBM2, TCP4, ERF003, GRF2, and GRF6, whereas Cluster 1 was characterized by highly connected TFs, including MIF1, HOX16, and CPC, and enrichment of hormone signaling, flavonoid biosynthesis, and protective barrier-related pathways. The hormone profiles further revealed stage- and tissue-associated changes, particularly the accumulation of ABA, IAA, JA, JA-Ile, and SA in 6WAG-Ha. These findings provide candidate genes and pathways for further investigation of coconut embryo and haustorium development. Functional validation will be required to determine the regulatory roles of the identified TFs and to clarify how hormone dynamics contribute to these developmental transitions.

## 4. Materials and Methods

### 4.1. Plant Materials

**Figure 9:**
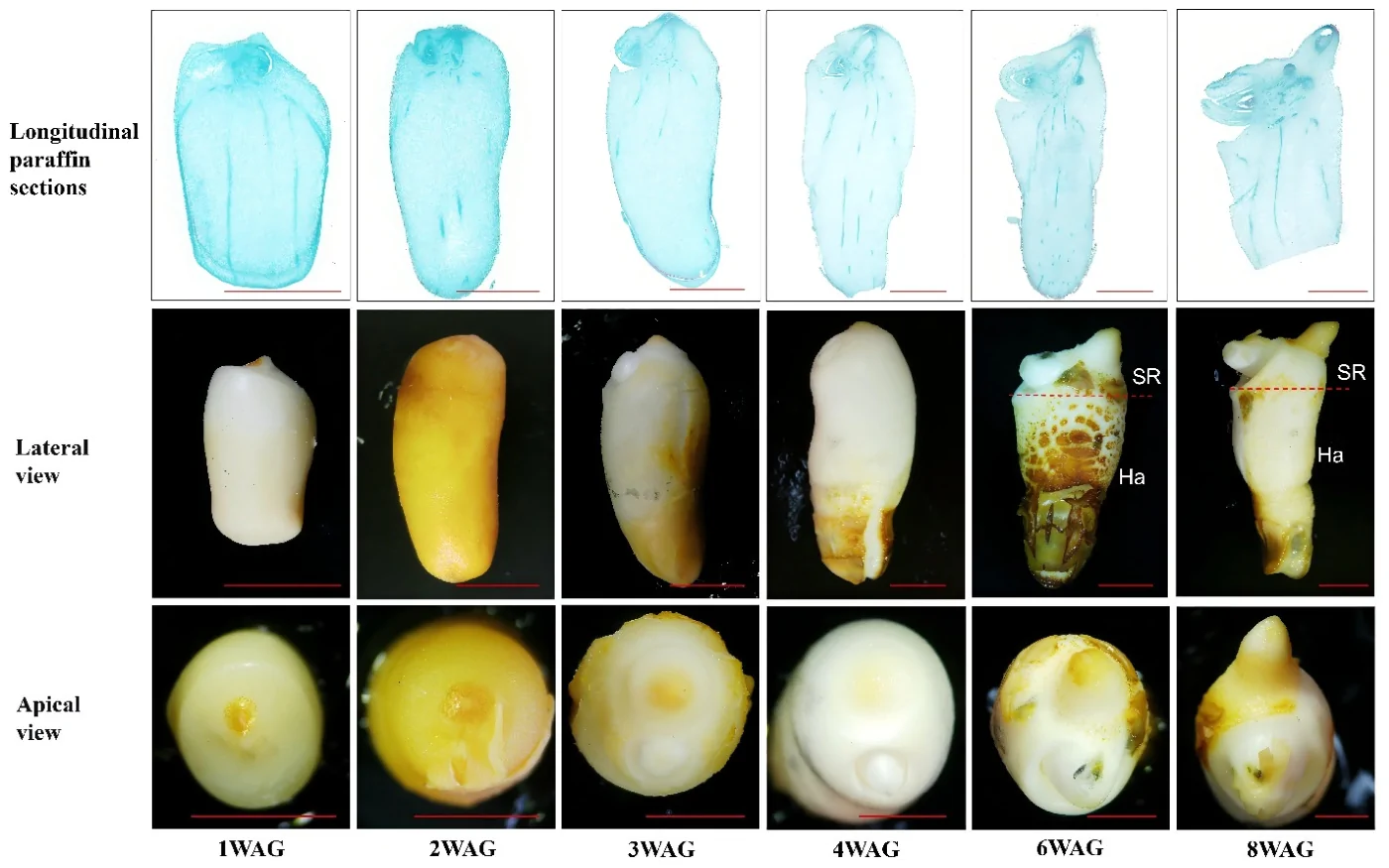
Morphological and anatomical changes during in vitro germination of coconut zygotic embryos. (**Top**), longitudinal paraffin sections; (**middle**), lateral views; (**bottom**), apical views of zygotic embryos at different stages of germination. Embryos progressively enlarged and developed from an externally intact state at 1WAG to surface rupture, plumule and radicle emergence, and subsequent elongation of the shoot, hypocotyl, and root system. At 2WAG, the plumule and radicle were clearly identifiable in sections, while by 3WAG the plumule had emerged from the cotyledonary tissues. Further culture resulted in progressive separation and elongation of the plumule, hypocotyl, and radicle. SR, the shoot/root part; Ha, the haustorium part. Scale bars: 5mm.

Zygotic embryos were collected from mature coconut fruits (12-month-old) from a 15-year-old coconut tree of the local variety ‘Hainan Tall’. Zygotic embryos were isolated and cultured onto germination medium (Y3 basic medium +40 g/L sucrose+ 4 g/L Phytagel+ 2.5 g/L Activated Charcoal). The cultures were incubated in the dark at 27 ± 1 °C. At 1WAG, zygotic embryos exhibited overall swelling with intact outer cotyledon epidermis. Paraffin sections revealed the initiation of the plumule, whereas the radicle was not yet anatomically detectable; thus, the plumule could not be identified by naked-eye observation. At 2WAG, distinct surface fissures appeared on the zygotic embryos, and part of the coleoptile became externally visible. Histological sections confirmed activation of germination, with both plumule and radicle primordia clearly distinguishable. At 3WAG, the outer tissues of zygotic embryos ruptured, and both plumule and radicle continuously expanded and elongated; the plumule fully broke through the enveloping cotyledonary tissues. Zygotic embryos collected at 2–3WAG were selected as explant material for plumule isolation: at this stage, plumules maintain a relatively low degree of cell differentiation for high callus-induction efficiency, and can be manually dissected under a stereomicroscope for efficient manipulation. By 4WAG of culture, plumule, hypocotyl and radicle had separated from cotyledon tissues and undergone further elongation. At 6WAG, paraffin section results revealed that the root system began to undergo further growth and development; in addition to the primary root, secondary roots also began to emerge. Complete outgrowth of shoot and root systems was observed from 6WAG to 8WAG (Figure 9). For more precise RNA sequencing and phytohormone determination in 6WAG to 8WAG, the shoot/root (upper part) and haustorium (lower part) were separated as WAG-SR and WAG-Ha.

### 4.2. RNA Extraction, Library Construction, and Sequencing

Three biological replicates were included for each developmental stage, with each replicate representing an independent biological sample. RNA sequencing was performed to characterize gene expression patterns during coconut zygotic embryo germination and haustorium development. Total RNA was extracted using Trizol reagent kit (Invitrogen, Carlsbad, CA, USA) according to the manufacturer’s instruction. RNA quality was assessed using an Agilent 2100 Bioanalyzer (Agilent Technologies, Palo Alto, CA, USA) and further evaluated by RNase-free agarose gel electrophoresis. After total RNA was extracted, eukaryotic mRNA was enriched by Oligo(dT) beads, while prokaryotic mRNAwas enriched by removing rRNA using the Ribo-Zero^TM^ Magnetic Kit (Epicentre, Madison, WI, USA). Then, the enriched mRNA was fragmented into short fragments using fragmentation buffer and reverse transcripted into complementary DNA (cDNA) with random primers. Second-strand cDNA was synthesized by DNA polymeraseI, RNaseH, deoxynucleoside triphosphate (dNTP), and buffer. Then, the cDNA fragments were purified with QiaQuick PCR extraction kit (Qiagen, Venlo, The Netherlands), end repaired, poly(A) added, and ligated to Illumina sequencing adapters. The ligation products were size selected by agarose gel electrophoresis, PCR amplified, and sequenced using Illumina HiSeq2500 by Gene Denovo Biotechnology Co. (Guangzhou, China).

### 4.3. RNA-Seq Data Processing and Differential Expression Analysis

Raw sequencing reads were filtered using fastp [39] (version 0.18.0) to remove adapter sequences, reads containing more than 10% of unknown nucleotides (N), and low-quality reads containing more than 50% bases with a quality score of Q-value ≤ 20. The remaining clean reads were first aligned against the ribosomal RNA (rRNA) database using Bowtie2 [40] (version 2.4.4), and rRNA-mapped reads were removed. The resulting clean reads were then mapped to the *Cocos nucifera* (tall) reference genome (cocos_nucifera_tall.genome.fa.gz) obtained from the Arecaceae Genome Database (AreCaceae-GDB; http://arecaceae-gdb.com) using HISAT2 [41] (version 2.1.0) with default parameters. Transcript assembly was performed using StringTie (v1.3.4), and gene expression abundance was quantified using Salmon (v1.10.3). Expression levels were represented as fragments per kilobase of transcript per million mapped reads (FPKM) and transcripts per million (TPM).

Differential expression analysis was performed using DESeq2 (v1.24) for pairwise comparisons. For comparisons between samples without biological replicates, edgeR (v3.12.1) was employed. Genes were considered differentially expressed when the false discovery rate (FDR) was <0.05 and the absolute fold change was ≥2.

### 4.4. KEGG Pathway Enrichment Analysis

KEGG pathway enrichment analysis was performed using the KEGG database (Release 117) to identify significantly enriched metabolic and signal transduction pathways among the differentially expressed genes (DEGs). A hypergeometric test was used to evaluate enrichment against the background of all genes annotated in the KEGG database. The resulting *p*-values were adjusted for multiple testing using the false discovery rate (FDR) correction. Pathways with an FDR ≤ 0.05 were considered significantly enriched.

### 4.5. Hierarchical Clustering of Core Developmental DEGs

To characterize the expression patterns of core developmental DEGs across coconut zygotic embryo germination, mean TPM values were calculated from the three biological replicates for each of the eight developmental stages/tissue groups (1, 2, 3, and 4WAG; 6WAG_SR; 6 WAG_Ha; 8WAG_SR; and 8WAG_Ha). The mean expression values were transformed using log_2_(TPM + 1), followed by gene-wise Z-score normalization (standardization across the eight developmental stages/tissue groups). Genes with missing Z-score values were excluded from subsequent clustering. Hierarchical clustering of genes was performed using the pheatmap package in R, with row clustering enabled and column clustering disabled. The resulting gene dendrogram was divided into four expression clusters using the cutree function with k = 4. Gene cluster assignments were subsequently combined with functional annotation information for downstream analyses.

### 4.6. Co-Expression Correlation Analysis

Co-expression analysis was performed using individual biological replicate TPM values from the eight developmental/tissue conditions, comprising three biological replicates per condition (24 samples in total). Averaged TPM values were excluded from the analysis. Gene expression values were transformed using log_2_(TPM + 1) before correlation analysis. Pearson correlation coefficients were calculated between each selected focal transcription factor (TF) and the other genes within the corresponding expression cluster using the cor.test function in R. Two-sided *p*-values were obtained for each correlation test and adjusted for multiple testing using the Benjamini–Hochberg (BH) procedure to control the false discovery rate (FDR). Significant co-expression relationships were defined using an absolute Pearson correlation coefficient (|r|) ≥ 0.90 and an FDR < 0.05. The resulting TF-centered co-expression networks were visualized using Cytoscape (version 3.3.0).

### 4.7. Phytohormone Profiling and Visualization

Phytohormone concentrations were determined in coconut zygotic embryos at eight developmental stages: 1, 2, 3, and 4 weeks after germination (WAG), and shoot/root (SR) and haustorial (Ha) tissues at 6 and 8 WAG. Twenty-one hormone-related metabolites representing auxins, ABA, jasmonates, SA, cytokinins, and gibberellins were analyzed, including IAA, ABA, JA, MeJA, JA-Ile, OPDA, SA, SAG, cytokinins, and GAs.

Approximately 10–20 embryos were pooled to obtain 1.5 g of sample for phytohormone analysis at each developmental stage. The collected tissues were immediately frozen in liquid nitrogen and stored at −80 °C until analysis. Due to limited sample availability and funding constraints, one pooled sample was analyzed per stage; therefore, independent biological replicates and statistical comparisons among developmental stages were not performed. Samples were ground and extracted with 1 mL methanol/water/formic acid (15:4:1, *v*/*v*/*v*). Extracts were evaporated under nitrogen, reconstituted in 100 µL 80% methanol, and filtered through 0.22 µm membranes. Hormones were quantified using UHPLC–ESI–MS/MS on an AB Sciex QTRAP 6500 system equipped with an ACQUITY UPLC HSS T3 C18 column. Chromatographic separation was performed using water and acetonitrile containing 0.04% acetic acid. Hormone quantification was conducted by MetWare using multiple-reaction monitoring (MRM), following the method of Pan et al. (2010) [42].

For visualization, hormone concentrations were independently standardized across the eight developmental stages using row-wise Z-score normalization. Hierarchical clustering was performed using Euclidean distance and complete linkage to group hormones according to their developmental profiles. Developmental stages were maintained in chronological order, and heatmaps were generated using the pheatmap package in R.

### 4.8. Data Analysis and Visualization

Gene expression data were analyzed from transcripts per million (TPM) values of three replicates. Heatmaps, UpSet plot, TFs analysis and lollipop charts for KEGG pathway analyses were generated using R [43] (version 4.1.0) and Rstudio [44] (version 1.4.1717). TF–TF–gene correlations were calculated in R, and the resulting interaction networks were visualized and constructed using Cytoscape.

## Data Availability

The RNA-seq data for this study can be found in the National Genomics Data Center-Genome Sequence Archive (GSA accession: CRA044758) (https://ngdc.cncb.ac.cn/gsa/s/ouxM56T5).

## Supplementary Materials

The following supporting information can be downloaded at: https://www.mdpi.com/article/10.3390/plants15213461/s1, Table S1: DEGs Statistics; Table S2: Core Developmental DEGs; Table S3: Cluster Annotation of Core Developmental DEGs; Table S4: TFs Analysis; Table S5: Phytohormone profiling.

## Abbreviations

ABA, abscisic acid; ARF, auxin response factor; cDNA, complementary DNA; DEGs, differentially expressed genes; dNTP, deoxynucleoside triphosphate; ESI, electrospray ionization; GA, gibberellin; GO, Gene Ontology; GRF, growth-regulating factor; Ha, haustorium; IAA, indole-3-acetic acid; JA, jasmonic acid; JA-Ile, jasmonoyl-isoleucine; KEGG, Kyoto Encyclopedia of Genes and Genomes; MeJA, methyl jasmonate; mRNA, messenger RNA; MS/MS, tandem mass spectrometry; OPDA, 12-oxo-phytodienoic acid; PCR, polymerase chain reaction; PCD, programmed cell death; RNA-seq, RNA sequencing; rRNA, ribosomal RNA; SA, salicylic acid; SAG, salicylic acid glucoside; SR, shoot/root; TFs, transcription factors; TPM, transcripts per million; tZR, trans-zeatin riboside; UHPLC, ultra-high-performance liquid chromatography; UPLC, ultra-performance liquid chromatography; WAG, weeks after germination; ZF-HD, zinc finger-homeodomain.
